# Host-Induced Gene Silencing of *SmDSR32* Enhances Wheat Defense Against *Sitobion miscanthi*

**DOI:** 10.3390/cimb48050523

**Published:** 2026-05-17

**Authors:** Jiahui Zhang, Xue Zhong, Mingxin Cao, Jiajing Xu, Mengchao Qin, Frédéric Francis, Lanqin Xia

**Affiliations:** 1 Institute of Crop Sciences/National Nanfan Research Institute, Chinese Academy of Agricultural Sciences (CAAS), Sanya 572024, China; zhangjiahui0806@163.com (J.Z.); zhongxue1124@163.com (X.Z.); caomingxin0112@163.com (M.C.); jjingxu27@163.com (J.X.); qinmengchao1995@163.com (M.Q.); 2 Functional and Evolutionary Entomology, Gembloux Agro-Bio Tech, University of Liege, Passage des Déportés 2, B-5030 Gembloux, Belgium; 3 State Key Laboratory of Crop Gene Resources and Breeding, Institute of Crop Sciences (ICS), Chinese Academy of Agricultural Sciences (CAAS), Beijing 100081, China; 4 College of Life Science and Agronomy, Zhoukou Normal University, Zhoukou 466001, China

**Keywords:** wheat (*Triticum aestivum* L.), grain aphid (*Sitobion miscanthi* F.), RNA interference (RNAi), salivary peptide, aphid control

## Abstract

The grain aphid, *Sitobion miscanthi*, poses a serious threat to cereal crops worldwide, leading to considerable yield losses and demanding annual insecticide applications during the grain-filling stage. As a sustainable alternative, we explored host-induced gene silencing (HIGS) targeting an aphid-specific gene. In this study, we identified *SmDSR32*, a novel gene encoding a salivary peptide in *S. miscanthi*, and validated its suitability for RNAi. Transgenic wheat lines expressing *SmDSR32-dsRNA* were generated. Aphids feeding on these lines showed a 20-fold reduction in *SmDSR32* transcript levels compared with controls. This silencing disrupted normal feeding behavior in electropenetrography (EPG) analyses, characterized by a 1.94-fold prolongation of intercellular probing and a 61% shortening of phloem ingestion. Consequently, aphid performance was severely compromised, with at least a 56.7% decrease in survival, a shortening of 5 days in lifespan, and a reduction of 9–10 individuals in aphid progeny production. Impressively, upon being transferred to wild-type plants, both the surviving aphids and their progeny sustained fitness deficits, with a 30% reduction in survival still observed in the first generation. These findings validate *SmDSR32* as a potent RNAi target and establish HIGS targeting essential salivary genes as a promising strategy for sustainable aphid management in wheat.

## 1. Introduction

Aphids (Hemiptera: Aphididae) rank among the most economically detrimental agricultural pests globally [1]. Their capacity for rapid population growth, diverse reproductive modes, and broad environmental tolerance collectively amplifies their destructive potential on cultivated crops [2,3,4]. As obligate phloem-feeding insects, high population densities of aphids result in substantial nutrient depletion from sieve tube elements, while their feeding activity facilitates the transmission of numerous plant-pathogenic viruses [5,6]. Despite documented ecological consequences and biodiversity threats associated with chemical control, its application persists as the prevailing approach, primarily due to its rapid efficacy in suppressing aphid outbreaks [7,8]. Consequently, developing alternative control strategies represents a critical priority to mitigate dependence on chemical pesticides. RNA interference (RNAi)-mediated gene silencing represents a strategy for enhancing crop resistance against diverse pests. This approach utilizes the host plant’s capacity to produce and deliver silencing RNAs (sRNAs) into invading pests, thereby targeting essential pest genes [9,10]. One prominent application of this RNAi principle is known as host-induced gene silencing (HIGS) [11]. Within the dsRNA-mediated RNAi pathway, host-derived dsRNAs are generated by transcribing complementary sequences of pest genes. These molecules subsequently target corresponding mRNA transcripts in pests, thereby decreasing functional protein synthesis [12]. Transgenic plant-mediated RNAi offers a protective and environmentally compatible approach for aphid management, with demonstrated efficacy in recent research [13,14,15].

Aphids utilize specialized piercing–sucking mouthparts, known as stylets, to extract phloem sap from host plants. This feeding process facilitates bidirectional substance exchange between the insect and its host [16]. Through stylet penetration, aphids acquire essential nutrients including carbohydrates, proteins, and nucleic acids from the phloem while simultaneously injecting salivary secretions into plant tissues [6,17]. The aphid saliva contains various bioactive components, with salivary proteins representing an important group of molecules. These proteins comprise diverse functional types, including digestive enzymes, immunomodulatory factors, and other bioactive components [18]. In piercing–sucking insects, salivary proteins are delivered into plants along with the gel and watery saliva secreted by salivary glands [19,20]. These proteins play crucial roles in facilitating digestion, metabolizing secondary compounds, detoxification, and modulating plant defense responses [21,22,23]. Salivary effectors can also enhance the post-transmission infections of arboviruses, including plasmodesmata-mediated cell-to-cell movement and phloem-mediated long-distance transport [24,25]. Aphid-excreted carbonic anhydrase facilitates intercellular movement of plant viruses during post-transmission infection [26]. The salivary protein glucose dehydrogenase (GLD) of *Myzus persicae* accelerates systemic movement of cucumber mosaic virus (CMV) via oxidizing plant sieve element occlusion (SEO) proteins, and GLD can be selected as a candidate target to impair the viral post-transmission infection process [27].

Notably, salivary protein composition exhibits both interspecific variation among aphid species and intraspecific plasticity depending on host plant species [28,29,30], reflecting the crucial role of salivary proteins in the ongoing co-evolutionary arms race between aphids and their host plants. Studies have implied that the genes encoding salivary proteins in aphids are potential candidates for aphid control in plants through plant-mediated RNAi. For instance, RNAi-mediated knockdown of the effector protein Armet transcript significantly impaired the feeding behavior of pea aphids (*Acyrthosiphon pisum*) [31]. The dsRNA targeting salivary effector *Sm9723* significantly reduced aphid survival rates and fecundity while impairing feeding behavior [32]. Similarly, silencing salivary effector *Sg2204* triggered a robust wheat defense response, further decreasing aphid survival, reproduction, and feeding efficiency. Notably, RNAi-mediated knockdown of *Sg2204* homologs across four additional aphid species consistently compromised their performance on host plants [33]. Furthermore, transgenic wheat expressing dsRNA targeting the salivary protein gene *SmDSR33* led to increased mortality and reduced fecundity of aphids [13].

In this study, we identified a novel putative salivary peptide gene, *SmDSR32*, in the grain aphid (*Sitobion miscanthi*) based on our previous transcriptomic analysis and dsRNA artificial diet assay [34]. Then we constructed RNAi vectors and generated stable transgenic wheat lines expressing *SmDSR32*-dsRNA. The effects on aphids were experimentally evaluated through bioassays, with a specific focus on alterations in feeding behavior, lifespan, population growth rate, and mortality. Surviving aphids showed effective gene silencing, and their offspring exhibited reduced fitness when subsequently reared on wild-type plants, suggesting a carry-over effect across generations.

## 2. Materials and Methods

### 2.1. Plants and Insects

In this study, the hexaploid wheat variety *Triticum aestivum* L. *cv* Zhengmai 7698 (ZM7698) was utilized. A total of 30 to 35 seeds from both wild-type and transgenic lines (32–64, 32–188 and 32–494) were sown in pots and cultivated in a climate chamber set at 22 °C, under a 16 h photoperiod, and with a relative humidity of 40–60%.

Grain aphids (*S. miscanthi*) were reared on two-leaf stage aphid-sensitive wheat seedlings in a controlled environment similar to growth conditions of plants. Aphids from a single clonal line were continuously reared on wheat seedlings for 24 h to produce synchronized nymphs. The adults were then removed, and their offspring were used for subsequent experiments. All experiments were conducted in a climate chamber under the conditions described above.

### 2.2. RNA Extraction and Characterization of SmDSR32

Total RNA was isolated from pooled aphid adults using TransZol Up reagent (TransGen Biotech, Beijing, China), and first-strand cDNA was synthesized with the FastKing RT Kit (Tiangen, Beijing, China). The full-length *SmDSR32* coding sequence was amplified using TransStart^®^ FastPfu DNA Polymerase (TransGen Biotech, Beijing, China) following the instructions. PCR products were sequenced by the Institute of Crop Sciences, Chinese Academy of Agricultural Sciences (Beijing, China). The theoretical isoelectric point (pI) and molecular weight (MW) of SmDSR32 were predicted using ExPASy (https://web.expasy.org/compute_pi/) (accessed on 18 October 2025). Transmembrane domains and signal peptide regions were analyzed using TMHMM 2.0 (http://www.cbs.dtu.dk/services/TMHMM/) (accessed on 18 October 2025) and SignalP 6.0 (http://www.cbs.dtu.dk/services/SignalP/) (accessed on 18 October 2025), respectively. Homologous sequences of *SmDSR32* in other aphid species were retrieved via the Basic Local Alignment Search Tool (BLAST) 2.17.0 searches against the National Center for Biotechnology Information (NCBI, https://www.ncbi.nlm.nih.gov/) database. A phylogenetic analysis of *SmDSR32* nucleotide sequences from twelve aphid species was performed using MEGA 11 software (www.megasoftware.net). The phylogenetic tree was constructed using the maximum likelihood method with 1000 bootstrap replicates to assess branch support.

### 2.3. Vector Construction and Wheat Transformation

To construct the RNAi vector targeting *SmDSR32*, a 411 bp gene-specific fragment was amplified using designed primers (Table S1). A 320 bp fragment of the *GFP* gene was used as a negative control in the aphid bioassay. Before vector construction, the targeted *SmDSR32* fragment was analyzed using BLAST to evaluate its specificity and potential off-target effects. The analysis compared the fragment’s sequences with the genomes of aphid non-target organisms, including wheat (taxid: 4565), human (taxid: 9606), and key aphid natural enemies. These natural enemies comprised *Chrysopa* (taxid: 76806), *Asaphes vulgaris* (taxid: 338020), Coccinellidae (taxid: 7080), Reduviidae (taxid: 27479), flower flies (taxid: 34680), Araneae (taxid: 6893), and Mantodea (taxid: 7504).

The PCR products were purified and inserted in an inverted repeat orientation into the *Spe* I/*Eco* RV and *Sac* I/*Hpa* I restriction sites of the pEasy-Blunt-Zero-*AdhI* vector, resulting in the hairpin construct Bzero-*SmDSR32*-*adhI*-*SmDSR32*. The resulting plasmid was digested with *Ssp* I and *Bsr* GI to release the expression cassette, which was subsequently recovered for transformation. The RNAi construct was driven by the maize *ubiquitin* (*Ubi*) promoter. Transformation was carried out via particle bombardment using immature embryos derived from the wheat cultivar ZM7698. Immature embryos (14–16 days post anthesis) were isolated and placed with scutellum side up on callus induction medium. Cultures were maintained in the dark at 25 ± 1 °C for 4 weeks to induce somatic embryogenesis. The induced somatic embryos were then transferred to Murashige and Skoog (MS) regeneration medium and incubated under a 16 h light/8 h dark photoperiod at 25 ± 1 °C for 2–3 weeks. Somatic embryos were induced on medium, and regenerated plants were grown and selected. Healthy transgenic seedlings were transferred to soil and cultivated to maturity for further analysis [35].

### 2.4. Quantitative Real-Time PCR Analysis

To assess the expression level of *SmDSR32* at various developmental stages, total RNA was extracted from grain aphid nymphs (four instars) and adults reared on susceptible wheat. For expression analysis in response to different host plants, adult aphids fed on transgenic and wild-type wheat were collected for RNA isolation. To investigate the relative expression level of *SmDSR32-dsRNA* in different transgenic lines, total RNAs were isolated from one-week-old leaves of wild type and different transgenic wheat plants.

First-strand cDNA synthesis was performed using standard protocols. Quantitative real-time PCR (qRT-PCR) was conducted with SYBR^®^ Green Real-time PCR Master Mix (Tiangen, Beijing, China) on an ABI 7300 Real-Time PCR System. The wheat *actin* gene, the aphid *actin* gene, and *ribosomal protein S27 A* (*Rps27A*) gene were used as internal reference genes, and *SmDSR32*-specific primers were employed for expression quantification (Table S1). All treatments had three biological replicates, and each replicate consisted of three technical replicates. Relative gene expression levels were calculated using the 2^−ΔΔCT^ method, with normalization to the geometric mean of the two reference genes [36].

### 2.5. Southern Blot Analysis

Genomic DNA was isolated from young T3 plant leaf tissues using the CTAB method following the protocol of Russell and Sambrook [37]. A total of 35 μg of genomic DNA was subjected to digestion with restriction enzyme *Hind* III overnight. The resulting DNA fragments were separated by electrophoresis on a 0.8% agarose gel prepared in 1× TBE buffer, run at 60 V for approximately 12–16 h. Following electrophoresis, DNA was transferred onto Hybond-N^+^ membranes (Amersham, UK). For probe labeling and hybridization, the digoxigenin (DIG) High Prime DNA Labeling and Detection Starter Kit I (Roche, Mannheim, Germany) was employed according to the manufacturer’s instructions. Prehybridization, hybridization, washing, and signal detection were all performed using the DIG system. DNA probes were generated using the primers SmDSR32S-F/R to amplify the target sequence for labeling (Table S1).

### 2.6. Aphid Bioassays

A single apterous adult grain aphid clonal lineage was reared on wheat seedlings in enclosed cages. To obtain synchronized offspring, the aphids were allowed to reproduce for a 24 h period, after which the newly produced nymphs were collected and transferred onto fresh transgenic wheat plants.

T3 generation homozygous wheat lines expressing *SmDSR32*-dsRNA were used to assess their effects on aphid survival and reproductive performance. At the 3- to 4-leaf stage, each wheat line was infested with 20 newly hatched first-instar nymphs. Aphid mortality was monitored daily throughout the experiment. For each wheat line, 10 individual plants were used as a biological replicate. The entire experiment was independently repeated three times to ensure reproducibility.

Life history traits of the aphids were calculated based on standard demographic parameters: the net reproductive rate, R_0_ = ∑l_x·_m_x_, the mean generation time, T = ∑ xl_x_m_x_/∑L_x_m_x_ the intrinsic rate of increase, r_m_ = (lnR_0_)/T, and the finite rate of increase, λ = e^rm^. In the equations, l_x_ is the surviving rate to a specific age x, and m_x_ is the number of newborn nymphs produced by per live adult for a specific age x [38].

### 2.7. Electrical Penetration Graph Technique Analysis

The probing and feeding behavior of apterous adult aphids on wheat was monitored using a Giga-8 DC EPG amplifier (EPG-Systems, Wageningen, Netherlands) within a Faraday cage. Synchronously aged adult aphids were first allowed to feed on either 32–188 transgenic wheat or control plants for 48 h. Following this preconditioning, aphids were starved for 2 h. Each aphid was then individually tethered via the dorsal thorax using water-soluble silver conductive paint to a flexible gold wire (18 μm in diameter, 2 cm in length). The aphids were subsequently placed on the adaxial surface of wheat leaves at the three-leaf stage. The opposite end of the gold wire (2 mm in diameter, 3 cm in length) was affixed to a copper wire using conductive silver glue, which was connected to the DC amplifier. A plant electrode was inserted into the soil to complete the electrical circuit. Each aphid’s EPG signal was continuously recorded for 8 h under illuminated conditions. A total of 8 individual recordings were obtained per treatment group. Signal analysis was performed using Stylet+a v01.30 software (EPG-Systems). Feeding behavior was interpreted based on waveform patterns, following the methodology established by [39,40]. The non-probing (np) waveform indicates that the stylet remains outside the leaf tissue. The pathway phase includes two distinct waveforms, waveform C, representing the intercellular movement of the stylet, and potential drops (pd), which correspond to brief intracellular punctures during this phase. Waveform G is associated exclusively with active sap uptake from xylem vessels. The phloem phase consists of two sequential components, E1, marking the initial secretion of saliva into the sieve elements, and E2, indicative of passive phloem sap ingestion. EPG recordings were further analyzed using the EPG-Excel data workbook developed by Sarria et al. [41].

### 2.8. Statistical Analysis

A two-tailed Student’s *t*-test was employed to assess statistical differences between wild-type and transgenic wheat lines. Significance levels were set at 1% or 5%. The standard error of the mean (SEM) was derived from three independent biological replicates for each treatment. In the EPG assays, mean values and corresponding SEMs were calculated based on individual aphid recordings, and statistical comparisons were performed using Student’s *t*-test. All results are expressed as mean ± SEM.

## 3. Results

### 3.1. Characterization of SmDSR32 Gene in Grain Aphids

**Figure 1:**
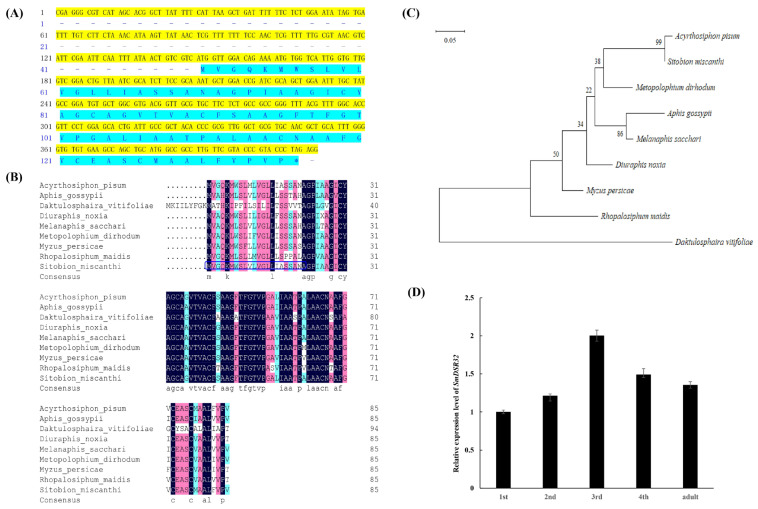
Characterization of SmDSR32 and generation of wheat RNAi lines. (**A**) The encoding sequence of SmDSR32 and its deduced amino acid sequence. The sequences selected for construction of the RNAi vector are highlighted in yellow. The deduced amino acid sequence is highlighted in blue. (**B**) Multiple sequence alignment of SmDSR32 protein and orthologs from other aphid species. The deduced amino acid sequences from eight aphid species include *Acyrthosiphon pisum* (XM_029487741.1), *Aphis gossypii* (XM_027982395.2), *Daktulosphaira vitifoliae* (XM_050677559.1), *Diuraphis noxia* (XM_015508058.1), *Melanaphis sacchari* (XM_025344765.1), *Metopolophium dirhodum* (XM_061021966.1), *Myzus persicae* (XM_022323304.1), and *Rhopalosiphum maidis* (XM_026966956.1). Black shades indicate identical amino acids. Pink shades indicate similar amino acid, and blue shades include the sequences with identical and similar residues. Signal peptide of SmDSR32 is highlighted with blue box. (**C**) Phylogenetic tree of SmDSR32 and its homologs from other aphid species constructed with the maximum likelihood method. Bootstrap supporting values (1000 replicates) are shown at the branch nodes. (**D**) The expression profile of *SmDSR32* in grain aphid at different development stages. The expression profiles of *SmDSR32* at different developmental stages of grain aphids fed on wheat. Values and error bars represent the mean and SEM of three independent biological replicates, each with a pool of 15 individual aphids.

Based on transcriptomic profiling and a dsRNA feeding assay, a candidate gene designated *SmDSR32*, which encodes a salivary peptide in the grain aphid, was identified [34]. The full-length sequence of this gene spans 261 bp and is predicted to encode an 86-amino-acid putative salivary peptide (Figure 1A). Bioinformatic analysis revealed that the SmDSR32 peptide has a predicted molecular weight (Mw) of 8254.84 Da and an isoelectric point (pI) of 5.74. Furthermore, the peptide is predicted to contain a signal peptide with a cleavage site between residues 22 and 23, alongside three transmembrane helices (Figure 1B and Figure S1).

To clarify the evolutionary relationships of this gene in different insect species, sequences of SmDSR32 counterparts in pea aphid (*A. pisum*), cotton aphid (*Aphis gossypii*), grape phylloxera (*Daktulosphaira vitifoliae*), Russian wheat aphid (*Diuraphis noxia*), sugarcane aphid (*Melanaphis sacchari*), rose-grain aphid (*Metopolophium dirhodum*), peach aphid (*M. persicae*), and corn aphid (*Rhopalosiphum maidis*) were obtained by BLAST search against NCBI database. The phylogenetic tree of the SmDSR32 peptide was constructed using MEGA 11 software, with the resulting analysis revealing its closest relationship to orthologs from the pea aphid (*A. pisum*) (Figure 1C). Furthermore, the BLAST analysis showed that the 411 bp fragment of *SmDSR32* did not share any contiguous 21-nucleotide sequences with known genetic sequences of aphid non-target organisms, including wheat, human, and key aphid natural enemies, indicating a low potential for off-target RNAi effects based on sequence homology [42].

The expression levels of *SmDSR32* across various developmental stages of the grain aphid were investigated using qRT-PCR, revealing that its transcript abundance accumulated to varying degrees throughout development (Figure 1D). The expression of *SmDSR32* reached its maximum in third-instar aphids, representing an approximate two-fold increase relative to the level observed in first-instar nymphs (Figure 1D).

### 3.2. In Planta RNAi of SmDSR32 in S. miscanthi via Transgenic Wheat Expressing dsRNA

**Figure 2:**
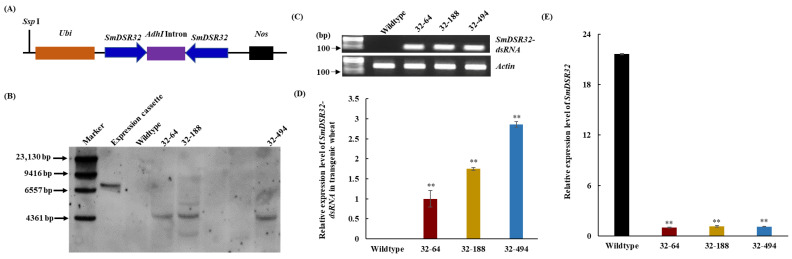
Generation and identification of wheat RNAi lines. (**A**) A schematic diagram of the *SmDSR32* expression cassette and the position of *Ssp* I restriction enzyme. (**B**) Southern blot analysis of the transgenic wheat lines. Genomic DNA was digested with *Ssp* I and hybridized with a *SmDSR32* gene fragment with the expression cassette digested with *Ssp* I as a positive control. (**C**) RT-PCR analysis of *SmDSR32-dsRNA* expression in different transgenic lines. *Actin* served as a reference transcript. (**D**) Relative expression levels of *SmDSR32-dsRNA* in different transgenic lines. (**E**) Relative expression levels of *SmDSR32* of grain aphid fed on wild-type and transgenic wheat lines. The expression level of *SmDSR32* in the adult aphids fed on wild-type and different transgenic wheat lines after inoculation of one-day-old newborn nymphs, respectively. Values and error bars represent the mean and SEM of three independent biological replicates, each with a pool of 15 surviving individual aphids (Student’s *t*-test, ** *p* < 0.01).

To assess the potential of *SmDSR32* as a target for dsRNA-mediated RNAi in aphid control, a 411 bp fragment of the gene was selected for hairpin RNAi vector construction (Figure 1A). A RNAi vector carrying *SmDSR32*-hairpin DNA was successfully engineered (Figure 2A). Following the transformation of immature wheat embryos (cultivar ZM7698), three independent transgenic lines (32–64, 32–188 and 32–494) were regenerated. Southern blot analysis confirmed the stable genomic integration of the transgene, with copy numbers varying from one to four (Figure 2B). The transcription levels and the relative expression levels of *SmDSR32-dsRNA* in different transgenic lines were investigated using RT-PCR and qRT-PCR. The result indicated that the transgenic line 32–494 had a higher dsRNA expression level than other two lines (Figure 2C,D). To evaluate whether the expression of *SmDSR32* in aphids was suppressed during feeding on transgenic wheat plants, one-day-old synchronized nymphs were individually transferred onto wild-type and transgenic wheat plants. Bioassay results revealed a significant suppression of *SmDSR32* expression (*p* < 0.01) in third-instar grain aphids fed on these transgenic lines compared to those on wild-type plants, confirming the successful induction of RNAi (Figure 2E).

### 3.3. The Impact of SmDSR32 Silencing on Feeding Behavior of S. miscanthi

**Figure 3:**
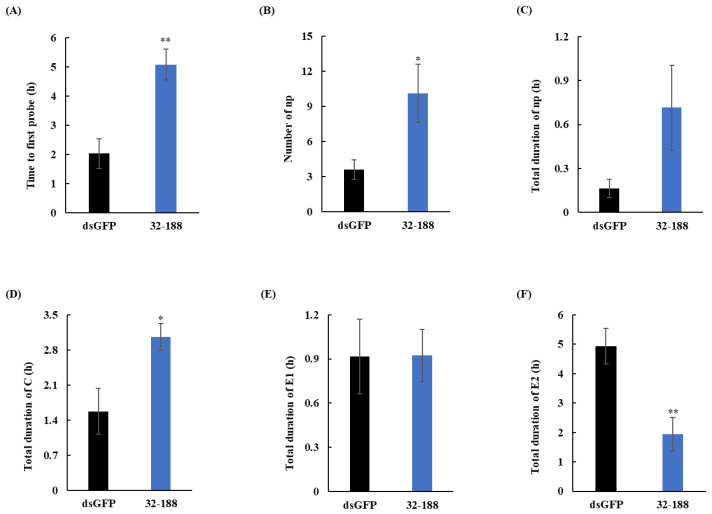
Feeding behavior of aphids fed on transgenic plants. (**A**–**F**) Representative parameters of aphid feeding behavior. Non-probing (np), stylet probing (C), intracellular stylet puncture (pd), phloem salivation (E1), and phloem ingestion (E2). Data shown are mean ± SEM. Asterisks above bars indicate significant differences between controls and treatments (Student’s *t*-test, * *p* < 0.05; ** *p* < 0.01).

To assess aphid feeding behavior, the transgenic wheat line 32–188 was selected for electropenetrography (EPG) assays. As illustrated in Figure 3A–F, *SmDSR32* silencing significantly impeded the feeding process. Compared to the control, aphids fed on transgenic plants exhibited a prolonged latency to first probe and spent significantly more time in non-probing (np) and C phases, indicating greater difficulty in locating suitable feeding sites (Figure 3A–D). Crucially, the duration of the sustained phloem ingestion (E2) phase was markedly shorter, demonstrating a compromised ability to acquire nutrients from the phloem (Figure 3F). The shortened E2 phase further reflects a decline in nutrient uptake efficiency. These findings collectively demonstrate that *SmDSR32* silencing disrupts key aspects of feeding behavior in grain aphids.

### 3.4. Silencing SmDSR32 via Transgenic Wheat Impairs Fitness of S. miscanthi

**Figure 4:**
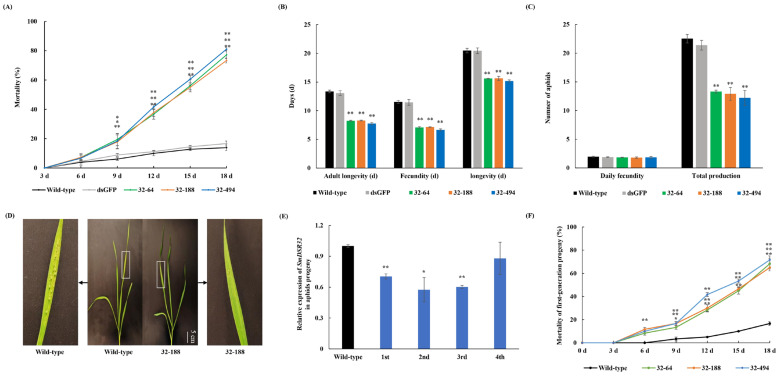
Fitness analysis of aphids fed on transgenic plants. (**A**) Mortality of aphids fed on wild-type and transgenic wheat lines. The mortality of aphids fed on wild-type and *dsSmDSR32* expression transgenic wheat lines. Twenty synchronous one-day-old nymphs were put into clip cages individually on transgenic and wild-type wheat plants. All experiments were repeated three times. Values and bars represent the mean ± SEM (Student’s *t*-test, * *p* < 0.05, ** *p* < 0.01). (**B**) The adult longevity, fecundity and the total longevity of aphids fed on transgenic wheat lines and wild-type control (Student’s *t*-test, * *p* < 0.05, ** *p* < 0.01). (**C**) The reproduction of aphids fed on transgenic wheat lines and the wild-type control. All experiments were repeated three times, each with 20 synchronous one-day-old nymphs. Values and bars represent the mean ± SEM (Student’s *t*-test, * *p* < 0.05, ** *p* < 0.01). (**D**) Phenotype of aphid resistance for wild-type (control) and 32-188 transgenic wheat plants. 10 synchronized one-day-old nymphs were placed on the leaf of plants. Photos were taken 12 days later. (**E**) The *SmDSR32* transcript levels of adult aphids were determined in fourth successive aphid generations. Black represents the wild type, and blue represents the fourth successive generation offspring. (**F**) The mortality of the first generation of the offspring of aphids fed on transgenic lines at different time points after being switched to wild-type plants. Values and bars represent the mean ± SEM (Student’s *t*-test, * *p* < 0.05, ** *p* < 0.01).

To evaluate the fitness consequences of *SmDSR32* silencing, key fitness parameters were compared between aphids reared on transgenic and wild-type wheat lines. Bioassays revealed a pronounced increase in mortality, exceeding 70% by 18 days after feeding (DAF) on transgenic plants (Figure 4A). While the adult preoviposition period (APOP) and total preoviposition period (TPOP) remained unaffected (Figure S2), aphids on transgenic wheat exhibited significantly reduced longevity, along with shorter adult lifespan and reproductive duration (*p* < 0.01) (Figure 4B). Consequently, a significant decline in total fecundity was observed across all three transgenic lines compared to the wild-type control (*p* < 0.01) (Figure 4C).

Collective analysis of key population parameters, including the net reproductive rate (R_0_), mean generation time (T), the intrinsic rates of increase (r_m_), the finite rate of increase (λ), and doubling time of the population (DT), revealed significant disparities between aphids reared on transgenic versus wild-type wheat lines (Table 1). Notably, R_0_, rₘ, and λ were all significantly suppressed in aphids feeding on the transgenic plants (*p* < 0.01) (Table 1).

### 3.5. Dietary RNAi Induces Persistent Fitness Effects Across Generations in S. miscanthi

To investigate whether the effects of *SmDSR32* silencing persist across generations, newborn nymphs from a parallel experiment were analyzed. Offspring derived from aphids fed on transgenic wheat exhibited constitutively suppressed *SmDSR32* expression and high mortality (Figure 4E,F), even when reared exclusively on wild-type plants. This suppression persisted from the first to the fourth generation (Figure 4E).

## 4. Discussion

Plant-mediated RNAi is widely regarded as a promising approach for engineering insect-resistant crops, notably for controlling wheat aphids, with important implications for global food security, human health, and agroecosystem sustainability [43,44,45]. HIGS, also known as host-mediated RNAi, involves the production of dsRNA molecules inside transgenic plants that target essential genes of insect pests. In our study, we expressed a hairpin RNA (hpRNA) complementary to the *SmDSR32* transcript in wheat. The plant’s intrinsic RNAi machinery processes this hpRNA into small interfering RNAs (siRNAs) or microRNAs (miRNAs), depending on the secondary structure of the dsRNA transcript. When aphids feed on the transgenic phloem, they ingest these processed siRNAs together with some unprocessed precursor dsRNAs [46]. Upon entry into the aphid gut cells, these RNA molecules trigger the insect’s own RNAi pathway which results in the silencing of the targeted gene [43]. Here, we successfully validated the salivary peptide gene *SmDSR32* of *S. miscanthi* as an effective RNAi target, and demonstrated that RNAi wheat based on HIGS strategy could significantly impact aphid feeding behavior and fitness (Figure 1, Figure 2, Figure 3 and Figure 4). Our results confirmed that aphids on RNAi lines showed significantly reduced *SmDSR32* expression levels, which led to a sharp decline in their survival rate, fecundity, and reproductive capacity (Figure 4A–C). This finding is consistent with numerous previous studies concluding that silencing key essential genes in insects, particularly those involved in feeding and digestion, can effectively impair pest survival and reproduction [13,32,33].

EPG analysis provided direct behavioral evidence for the physiological mechanism underlying *SmDSR32* silencing. Aphids feeding on RNAi plants exhibited prolonged durations of non-probing (np) and intercellular probing (C-wave) and substantially shortened durations of passive phloem sap ingestion (E2-wave) (Figure 3A–F). The E2 reduction is a direct measure of impaired sap uptake from sieve elements. This indicates that silencing *SmDSR32* severely impairs the aphid’s ability to locate and establish a rewarding feeding site. The aphids required more time for probing to find a suitable feeding location but struggled to maintain sustained sap ingestion. Since SmDSR32 is a putative salivary peptide, we hypothesize that it is secreted into sieve elements during the E1 (salivation) phase and normally facilitates sustained sap flow—perhaps by suppressing sieve tube occlusion or by modulating plant defense responses such as callose deposition [21,47]. These results provide direct in vivo behavioral evidence linking a specific salivary gene to phloem ingestion efficiency. This alteration in feeding behavior closely correlates with the observed reduction in aphid fitness, further underscoring the critical role of salivary components in aphid–plant interactions [2,48,49,50].

Furthermore, not only did the aphids directly feeding on transgenic plants incur fitness costs, but their offspring also continued to exhibit reduced fitness, even when reared on wild-type plants. By the fourth generation, *SmDSR32* expression returned to normal (Figure 4E,F). This transgenerational RNAi effect has been reported in aphids and likely results from persistent siRNAs in the germline or early embryos [51]. The absence of an RNA-dependent RNA polymerase (RdRP) in insect limits amplification, which explains why the effect fades without continuous dsRNA exposure. This multiplicative effect significantly enhances the practical application potential of HIGS technology, as even short-term or partial feeding could exert long-term, profound inhibitory impacts on pest populations [52,53].

Selecting an insect-specific salivary peptide gene *SmDSR32* as the target minimizes potential risks to humans and other non-target organisms, because the target fragment did not share any contiguous three 21-nucleotide sequences with the known genetic sequences of these organisms [42]. Moreover, the world’s first sprayable RNA-based biopesticide “Calantha” marks a milestone breakthrough in the field of crop protection. Field trials have demonstrated that Calantha not only effectively controls pest populations but also exhibits rapid degradation and high environmental safety, with no significant impact on non-target organisms [54]. Our transgenic HIGS approach provides an alternative delivery platform that could be integrated with sprayable dsRNA or stacked with other RNAi targets.

While our results are promising, the exact molecular function of SmDSR32—whether it binds to specific plant receptors, modifies sieve tube proteins, or inhibits callose deposition—requires direct biochemical investigation. In addition, translating this technology into practical applications requires consideration of potential challenges. For instance, continuous monitoring is needed to assess whether aphid populations could develop RNAi resistance through target gene mutations [55]. Furthermore, stacking *SmDSR32* dsRNA with dsRNAs targeting other genes could build a more robust and durable multi-gene pest resistance system [56]. Additionally, the combination of entomopathogenic fungus *Metarhizium anisopliae* and virus-induced gene silencing-RNAi (VIGS-RNAi) is also an efficient strategy for managing herbivorous pests [57]. Finally, evaluating the performance of our transgenic wheat lines under diverse field conditions across different wheat genetic backgrounds will be essential before commercial deployment.

## 5. Conclusions

In this study, we generated transgenic wheat lines expressing *SmDSR32*-dsRNA using a HIGS approach. EPG analysis revealed that aphids fed on transgenic plants exhibited prolonged non-probing and pathway phases (np, C) and drastically shortened phloem ingestion (E2). Silencing *SmDSR32* significantly reduced aphid survival, fecundity, and population growth. Furthermore, the suppressed gene expression and high mortality persisted in offspring even without continuous exposure to transgenic plants.

In conclusion, our study demonstrates that *SmDSR32* is a highly effective RNAi target for controlling the grain aphid, *S. miscanthi*. This lays a solid theoretical foundation and provides technical support for developing novel and sustainable wheat aphid management strategies. Future work should focus on elucidating the molecular interaction of SmDSR32 with host defense regulators, developing multi-target RNAi stacks, testing field performance across different wheat cultivars, and integrating HIGS with sprayable RNAi biopesticides.

## Figures and Tables

**Table 1:** Life table parameters of aphids fed on wild-type and different transgenic wheat lines.

Parameters	Wild-Type	32–64	32–188	32–494
R_0_	21.27 ± 0.52	10.21 ± 0.36 **	9.50 ± 0.41 **	8.99 ± 0.56 **
T	14.49 ± 0.30	13.51 ± 0.21	13.23 ± 0.28 *	13.10 ± 0.24 *
r_m_	0.21 ± 0.01	0.17 ± 0.00 **	0.17 ± 0.01 **	0.17 ± 0.01 *
λ	1.24 ± 0.01	1.19 ± 0.01 **	1.19 ± 0.01 **	1.18 ± 0.01 **
DT	3.29 ± 0.08	4.04 ± 0.11 **	4.10 ± 0.16 *	4.17 ± 0.20 *

All data are expressed as means ± SEM based on 3 repeated experiments. R_0_, net reproductive rate; r_m_, the intrinsic rate of increase; λ, the finite rate of increase; T, the mean generation time; DT, Doubling time (day). Student’s *t*-test, *n* = 3, * *p* < 0.05, ** *p* < 0.01.

## Data Availability

The original contributions presented in this study are included in the article/Supplementary Material. Further inquiries can be directed to the corresponding authors.

## Supplementary Materials

The following supporting information can be downloaded at: https://www.mdpi.com/article/10.3390/cimb48050523/s1, Figure S1: The predicted signal peptide and transmembrane helix of SmDSR32; Figure S2: The longevity of different stages, adult preoviposition period (APOP) and total preoviposition period (TPOP) of aphids fed on transgenic lines and wild-type control; Table S1: Primers used in this study.
