# Supplementary material for: Host-Induced Gene Silencing of SmDSR32 Enhances Wheat Defense Against Sitobion miscanthi
Source: Curr Issues Mol Biol. 2026 May 17;48(5):523. doi: 10.3390/cimb48050523 (PMC13206040; doi:10.3390/cimb48050523)
Supplement: Supplementary file 1 [file cimb-48-00523-s001.zip › cimb-4231316-supplementary.pdf]

Supplementary

Contents

Supplementary Figure S1. The predicted signal peptide and transmembrane helix of SmDSR32.

Supplementary Figure S2. The longevity of different stages, adult preoviposition period (APOP) and total preoviposition period (TPOP) of aphids fed on transgenic lines and wild-type control.

Supplementary Table S1. Primers used in this study.

(A)

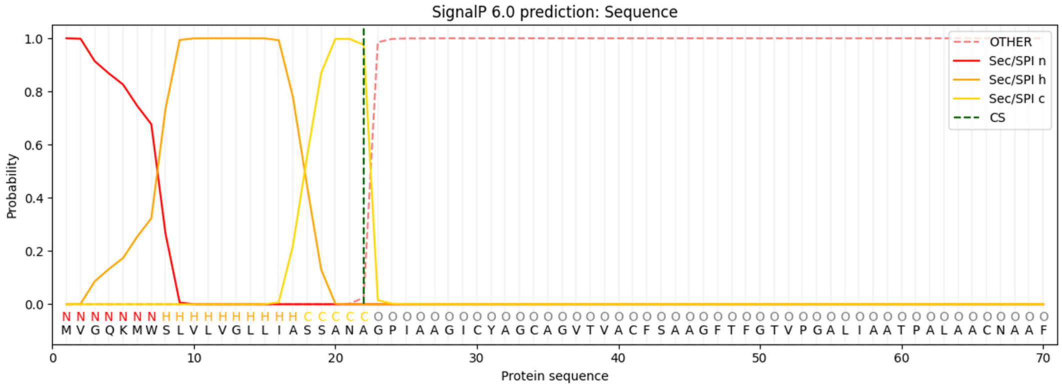

(B)

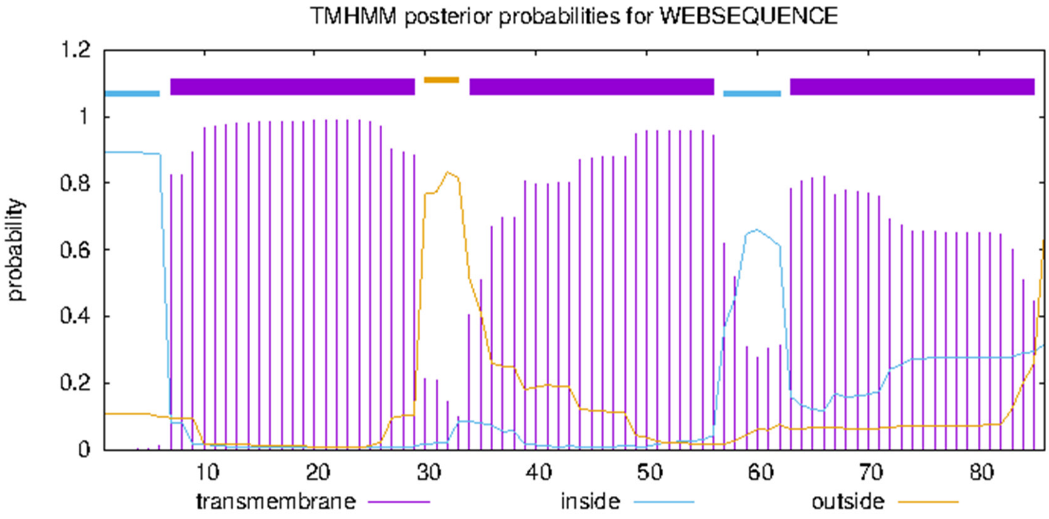

Supplementary Figure S1. The predicted signal peptide and transmembrane helices of SmDSR32.

(A) The predicted signal peptide of SmDSR32 using SignalP. The cleavage site is between residues $A^{20}N^{21}A^{22}$-$G^{23}P^{24}$.

(B) The predicted transmembrane helices of SmDSR32 using TMHMM. Three transmembrane helices were identified.

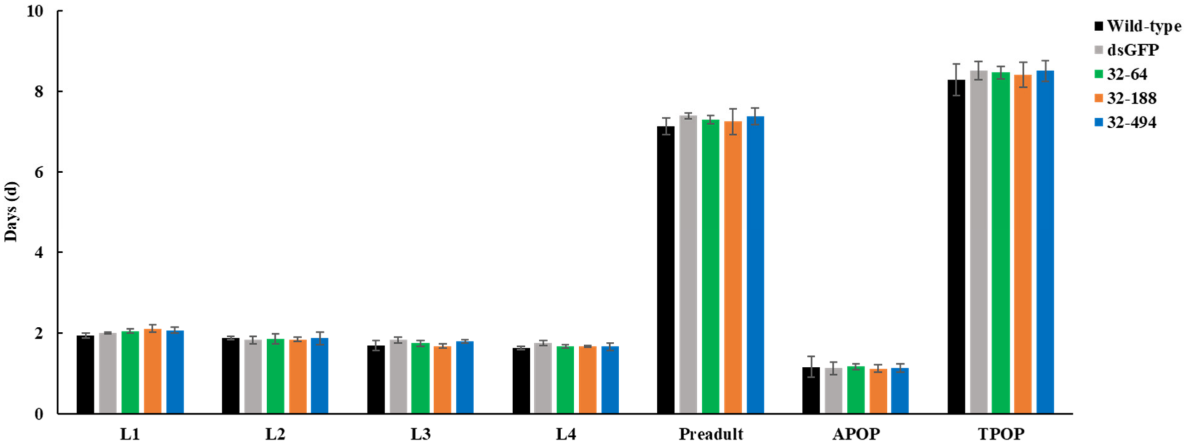

Supplementary Figure S2. The longevity of different stages, adult preoviposition period (APOP) and total preoviposition period (TPOP) of aphids fed on transgenic lines and wild-type control.

L1-L4: first-instar to fourth-instar aphids. APOP: the duration from adult emergence to first reproduction; TPOP: the duration from birth to first reproduction. All experiments were repeated three times, each with 20 synchronous one-day-old nymphs. Values and bars represent the mean ±SEM (Student's *t*-test, $^{*}P<0.05$, $^{**}P<0.01$).

Table S1. Primer sets used in this study

Primer	Sequence (5′ to 3′)	Use of PCR products	Annealing (℃)	Fragment size (bp)
SmDSR32c-F	ATGGTTGGACAGAAAATGTGGTC	Full length cDNA cloning	57	261
SmDSR32c-R	CTAGGGTACGGGTACGAACA			
SmDSR32S-F	AGGTCGACTCTAGAGGATCCCCGGGGGATCCACTAGTCGAGGGCGTCATAGCACGG	Construct vector for RNAi	59	472
SmDSR32E-R	GGCCTCCCAGATCGATTCGATATCCCTCTAGGGTACGGGTACGAAC			
SmDSR32H-F	AGGGGCAGACTCCCGTTTGTTAACCCTCTAGGGTACGGGTACGAAC	Construct vector for RNAi	59	460
SmDSR32S-R	TGAACGATCGGGGAAATTCGAGCTCCGAGGGCGTCATAGCACGG			
qactin-F	CGGTTCAAAAACCCAAACCAG	Internal control of qRT-PCR for aphid	56	260
qactin-R	TGGTGATGATTCCCGTGTTC			
Rps27-F	TGTGAAGACGTTGACTGGGAAA	Internal control of qRT-PCR for aphid	57	114
Rps27-R	CGTTGCTGATCCGGAGGAATAC			
SmDSR32S-F	CGACGAGTCTAACGGACACC	Southern blot	58	456
SmDSR32S-R	CCCTAACCATGGACCGGAAC			
SmDSR32q-F	GTGGTCATTGGTGTTGGTCG	Detect *SmDSR32* in aphids	57	139
SmDSR32q-R	AACGGTGCCAAACGTAAACC			
Ubi-F	GCCTTCATACGCTATTTATTTGCTTGGTAC	Detect *SmDSR32* dsRNA in transgenic wheat	60	659
ADH1-R	GGAGTCTGCCCCTAAGACAGATAAGC			
ADH1-F	CTGGGAGGCCAAGGTATCTAATCAGC	Detect *SmDSR32* dsRNA in transgenic wheat	60	619
Nos-R	CGCAAGACCGGCAACAGGATTC			
